# Still too much delay in recognition of autism spectrum disorder

**DOI:** 10.1017/S2045796021000822

**Published:** 2022-01-11

**Authors:** Bonati Maurizio, Massimo Cartabia, Antonio Clavenna

**Affiliations:** Department of Public Health, Istituto di Ricerche Farmacologiche Mario Negri IRCCS, Milan, Italy

Cohort studies have consistently reported trends in autism spectrum disorder (ASD) diagnosis with a considerable increase in prevalence and incidence over the last two decades (Lyall *et al*., 2016). Although the descriptive epidemiological studies of ASD, mainly focused on children and in high-income countries (Chiarotti and Venerosi, 2020), are affected by numerous methodological limitations all are in agreement with the increase. The rising incidence rates have been attributed differently from one country to another to different factors such as increased awareness of ASD, changes in diagnostic tools and criteria, lowered stigma and improved health service organisation (Hodges *et al*., 2020). To define the size and slope of the increase is useful to determine whether the prevalence has stabilised. Thus, we examined the time trends of cumulative incidence of ASD over the last 20 years in a large Italian population.

## Methods

This study was reported following the Strengthening the Reporting of Observational Studies in Epidemiology (STROBE) reporting guideline. No statistical tests were conducted in this study. Data on children born in 2000–2017, resident in Lombardy Region until 31/12/2020, and diagnosed with ASD (International Statistical Classification of Diseases, Ninth Revision [ICD-9] code 2990*) were retrieved from the administrative health databases of Italy's Lombardy Region. The Lombardy Region is one of the largest Italian regions; it is situated in the north and is one of the most prominent from the socioeconomic point of view. In Lombardy Region, healthcare is provided free up to the age of 14. The used databases have been previously validated and extensively used for epidemiological studies.

For each child included in the study, we defined an observational period starting from the first birthday and ending on the day before the birthday of 2020. Then, ASD case status (incidence case) was determined using the first time that for a child was reported ASD condition in one of the health administrative regional databases (hospitalisations or specialist visits) during the observational period.

Extracted information included date of birth, gender and day of the first diagnosis. Thus, for each birth cohort from 2000 to 2017, we calculated cumulative ASD incidence as the cumulative sum of the number of new cases of ASD for each age divided by the total number of children born in the same cohort. Lifetime cumulative incidence refers to individuals with a reported ASD condition in a period of years of life considered.

## Results

We included in this study 1 323 792 children (681 048 boys and 642 744 girls) born between 2000 and 2017 and resident in Lombardy Region until 31/12/2020. The number of children diagnosed with ASD between 1 and 19 years of age was 8105 (0.61%), 6627 (0.97%) boys and 1478 (0.23%) girls. Table 1 shows the cumulative incidence values for the 18 birth cohorts. The maximum value was 1.06% (95% CI 0.98–1.13%) at age 4 years for persons born in 2015. This class of age showed the major increase in the cumulative incidence (+0.97%) going from 0.09% (95% CI 0.07–0.11%) for the 2001 cohort to 1.06% (95% CI 0.98–1.13%) for the 2015 cohort.

**Table 1.** Cumulative incidence of autism spectrum disorder through 2020 by age per 100 persons (%) born between 2000 and 2017 (95% CI) in Lombardy Region, Italy

Birth year	Age 2	Age 4	Age 6	Age 8	Age 10	Age 18
2000	0.04 (0.03–0.06)	0.13 (0.10–0.16)	0.16 (0.13–0.19)	0.18 (0.15–0.21)	0.21 (0.18–0.25)	0.33 (0.29–0.37)
2001	0.02 (0.01–0.04)	0.09 (0.07–0.11)	0.13 (0.10–0.15)	0.17 (0.14–0.20)	0.20 (0.17–0.23)	0.34 (0.30–0.39)
2002	0.04 (0.02–0.05)	0.13 (0.10–0.15)	0.19 (0.16–0.22)	0.23 (0.20–0.27)	0.28 (0.24–0.31)	
2003	0.06 (0.04–0.07)	0.13 (0.11–0.16)	0.19 (0.16–0.23)	0.24 (0.21–0.28)	0.29 (0.25–0.33)	
2004	0.05 (0.03–0.06)	0.16 (0.13–0.19)	0.22 (0.18–0.25)	0.29 (0.25–0.33)	0.34 (0.30–0.38)	
2005	0.05 (0.04–0.07)	0.17 (0.14–0.20)	0.26 (0.22–0.30)	0.32 (0.28–0.36)	0.37 (0.33–0.41)	
2006	0.06 (0.05–0.08)	0.19 (0.16–0.22)	0.30 (0.27–0.34)	0.37 (0.33–0.41)	0.44 (0.40–0.49)	
2007	0.07 (0.05–0.09)	0.23 (0.19–0.26)	0.30 (0.26–0.34)	0.38 (0.34–0.43)	0.47 (0.42–0.52)	
2008	0.07 (0.05–0.09)	0.23 (0.20–0.27)	0.33 (0.29–0.36)	0.42 (0.38–0.46)	0.51 (0.46–0.56)	
2009	0.07 (0.05–0.09)	0.24 (0.21–0.27)	0.37 (0.32–0.41)	0.47 (0.43–0.52)	0.55 (0.50–0.60)	
2010	0.10 (0.08–0.13)	0.30 (0.25–0.34)	0.42 (0.37–0.47)	0.54 (0.49–0.60)		
2011	0.15 (0.12–0.18)	0.43 (0.38–0.47)	0.64 (0.58–0.70)	0.77 (0.71–0.84)		
2012	0.22 (0.18–0.25)	0.66 (0.60–0.72)	0.90 (0.83–0.97)			
2013	0.32 (0.28–0.36)	0.81 (0.74–0.88)	1.04 (0.96–1.11)			
2014	0.35 (0.31–0.39)	0.97 (0.90–1.05)				
2015	0.42 (0.37–0.46)	1.06 (0.98–1.13)				
2016	0.37 (0.33–0.42)					
2017	0.28 (0.24–0.32)					

**Fig. 1.**
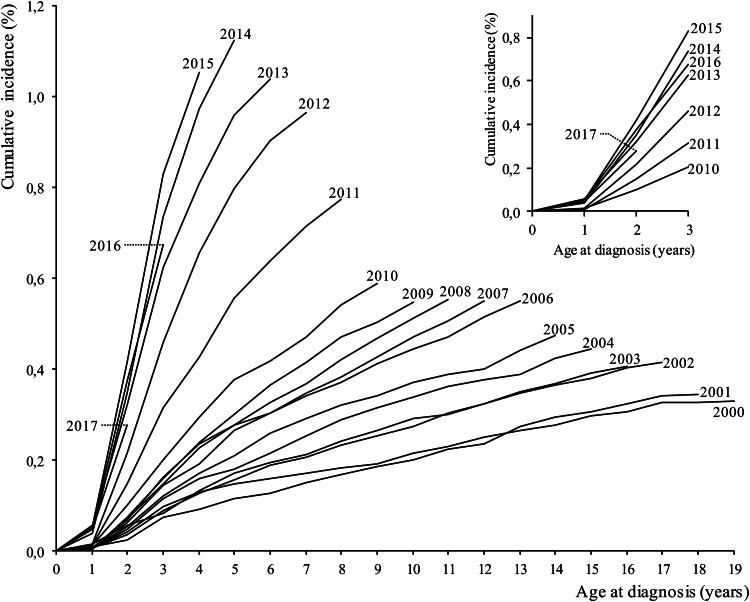
Each curve in the main body of the figure corresponds to the autism spectrum disorder cumulative incidence through 2020 among person in each birth cohort (beginning 2000, bottom curve). The inset is a close-up view of the ASD cumulative incidence through age 3 years for each birth cohort.

The 2-year lifetime cumulative incidence ranges between 0.02% (95% CI 0.01–0.04%) for the 2001 cohort and 0.42% (95% CI 0.37–0.46%) for the 2015 cohort (Fig. 1). The 2-year lifetime cumulative incidence value achieved since the 2013 birth cohort is similar to that of 2000 at 18 years of age. These trends are particularly marked in all cohorts with a boys/girls cumulative incidence ratio ranging from 3.35 in 2000 birth cohort to 6.00 in that of 2010 (see online Supplementary Table S1). A continuous increase in the slope of cumulative incidence is observable for all the studied cohorts but it is starting from those of the last decade that the shift is more marked (Fig. 1).

## Discussion

In recent years, cases of autism have risen everywhere in the world (Baxter *et al*., 2015). About one in 105 children born in 2015 in the Italian Lombardy Region was identified with ASD by age 4. This is a 75% increase from children born in 2009. These trends are consistent with other reported birth cohort analyses, although the national slope and profile of the cumulative incidence curve show different trajectories (Schendel and Thorsteinsson, 2018; Segev *et al*., 2019; Rah *et al*., 2020; Cybulski *et al*., 2021; Sasayama *et al*., 2021). However, also these findings suggest that ASD cumulative incidence has not stabilised, and the appearance of ASD condition in the administrative health databases with increasing age indicates a delay in the interception of ASD status. That ASD is more than four times common among boys than among girls is generalisable, not so much the effective rate of ASD as long as the curve of incidence will reach a plateau, and the value of the rate will be stable between the new births cohorts. The increasing rate of the young cohorts and with the age in older ones suggests the improvement in making an early diagnosis than in the past. Although it is difficult to make accurate predictive estimates, early cumulative incidence could exceed 1.1% (one in 91 new births). Expanding public awareness may have contributed to the increased nationwide incidence of ASD, as well as non-etiological factors (e.g. accessibility to services, diagnostic criteria). However, the results highlight the need for quickly improving effective initiatives tracking appropriately trends in prevalence and incidence of ASD (e.g. national registries, surveillance programmes) (Fombonne *et al*., 2021), and covering the whole ASD care, throughout the life span, for a growing target population. A public challenge for everyone and not just healthcare.

## Data Availability

The dataset supporting the conclusions of this study is available from the corresponding author upon request.

## Supplementary material

For supplementary material accompanying this paper visit https://doi.org/10.1017/S2045796021000822. click here to view supplementary material
