# Supplementary material for: Still too much delay in recognition of autism spectrum disorder
Source: Epidemiol Psychiatr Sci. 2022 Jan 11;31:e1. doi: 10.1017/S2045796021000822 (PMC8786613; doi:10.1017/S2045796021000822)
Supplement: Supplementary file 1 [file S2045796021000822sup001.docx]

**SUPPLEMENT**

**Table. Boys/Girls Cumulative Incidence Ratio of Autism Spectrum Disorder Through 2020 by Age per 100 Persons (%) Born Between 2000 and 2017 (95% CI) in Lombardy Region, Italy**

| Birth Year | 1 | 2 | 3 | 4 | 5 | 6 | 7 | 8 | 9 | 10 | 11 | 12 | 13 | 14 | 15 | 16 | 17 | 18 | 19 |
| --- | --- | --- | --- | --- | --- | --- | --- | --- | --- | --- | --- | --- | --- | --- | --- | --- | --- | --- | --- |
| 2000 | 1.40 | 2.18 | 4.25 | 3.79 | 3.88 | 4.28 | 4.42 | 4.33 | 4.17 | 4.75 | 4.95 | 4.80 | 4.79 | 4.39 | 4.06 | 3.85 | 3.47 | 3.34 | 3.35 |
| 2001 | 1.26 | 1.90 | 2.71 | 3.59 | 4.03 | 3.74 | 4.27 | 4.35 | 4.46 | 4.66 | 5.10 | 4.74 | 4.86 | 4.93 | 5.15 | 4.84 | 4.86 | 4.79 |  |
| 2002 | 0.31 | 2.56 | 3.21 | 3.73 | 3.77 | 4.10 | 4.37 | 4.65 | 4.40 | 4.50 | 4.72 | 4.82 | 4.81 | 4.94 | 4.64 | 4.64 | 4.72 |  |  |
| 2003 | 2.51 | 3.46 | 3.30 | 3.97 | 4.80 | 5.04 | 4.48 | 4.97 | 4.77 | 4.22 | 4.41 | 4.31 | 4.05 | 4.00 | 4.02 | 3.95 |  |  |  |
| 2004 | 2.89 | 7.71 | 3.75 | 3.56 | 3.82 | 4.20 | 4.59 | 4.55 | 4.36 | 4.77 | 4.64 | 4.62 | 4.58 | 4.38 | 4.12 |  |  |  |  |
| 2005 | 3.73 | 3.85 | 4.79 | 4.16 | 4.21 | 4.25 | 4.57 | 4.53 | 4.91 | 4.97 | 5.11 | 5.20 | 4.92 | 4.65 |  |  |  |  |  |
| 2006 | 4.70 | 8.46 | 6.33 | 5.86 | 5.10 | 5.56 | 5.99 | 6.01 | 6.07 | 5.99 | 5.60 | 5.69 | 5.70 |  |  |  |  |  |  |
| 2007 | - | 6.59 | 5.88 | 4.35 | 4.51 | 4.55 | 4.83 | 5.13 | 5.18 | 5.03 | 5.13 | 4.59 |  |  |  |  |  |  |  |
| 2008 | 1.42 | 2.91 | 3.76 | 3.87 | 4.20 | 4.57 | 4.59 | 4.61 | 4.59 | 4.68 | 4.89 |  |  |  |  |  |  |  |  |
| 2009 | 0.47 | 3.40 | 4.19 | 3.93 | 4.00 | 3.92 | 3.79 | 4.05 | 4.05 | 4.05 |  |  |  |  |  |  |  |  |  |
| 2010 | 2.80 | 4.83 | 4.79 | 5.43 | 5.09 | 5.26 | 5.59 | 5.95 | 6.00 |  |  |  |  |  |  |  |  |  |  |
| 2011 | 2.83 | 3.10 | 4.33 | 4.67 | 4.35 | 4.93 | 4.26 | 4.26 |  |  |  |  |  |  |  |  |  |  |  |
| 2012 | - | 4.07 | 4.05 | 4.02 | 3.99 | 4.21 | 4.39 |  |  |  |  |  |  |  |  |  |  |  |  |
| 2013 | 3.20 | 3.85 | 2.69 | 3.92 | 3.97 | 3.99 |  |  |  |  |  |  |  |  |  |  |  |  |  |
| 2014 | 4.21 | 3.60 | 3.75 | 3.82 | 3.69 |  |  |  |  |  |  |  |  |  |  |  |  |  |  |
| 2015 | 2.91 | 4.21 | 3.83 | 3.81 |  |  |  |  |  |  |  |  |  |  |  |  |  |  |  |
| 2016 | 4.48 | 3.90 | 3.62 |  |  |  |  |  |  |  |  |  |  |  |  |  |  |  |  |
| 2017 | 3.10 | 3.44 |  |  |  |  |  |  |  |  |  |  |  |  |  |  |  |  |  |
